# Game modelling and stability strategy research on distributed leadership pattern: A tripartite evolutionary game perspective

**DOI:** 10.1371/journal.pone.0271721

**Published:** 2022-07-20

**Authors:** Ledi Lyu, Zeguang Cui, Haomin Zhang

**Affiliations:** School of Management, Shanghai University, Shanghai, PR China; University of Electronic Science and Technology of China, CHINA

## Abstract

Distributed leadership pattern has been a topic of growing interest in recent years, recognizing that much remains to be known about this phenomenon. The research on distributed leadership acknowledges that responsibility and power are not exclusively limited to one formal leader, but are distributed between formal and informal leaders. The decision-making behavior of team members plays a vital role in optimizing cooperation and team performance. Nevertheless, little attention is paid to investigating the underlying mechanisms about how people in a team cooperate to initiate effective interactions and enhance team performance. Game theory offers a comprehensive analysis of rational behavior under the circumstances of strategic interdependence. By organizing the formal leader, the informal leader, and the ordinary employee in a team, this study constructs a tripartite evolutionary game model and analyzes the internal mechanism of distributed leadership patterns. The study finds that the equilibrium of the three parties is affected by multiple factors. The simulation results reveal that the empowerment of the formal leader to the informal leader is indispensable to promoting optimal cooperation and team performance in distributed leadership patterns. These findings have theoretical implications for the distributed leadership literature and managerial implications for practitioners.

## 1. Introduction

Since modern teams are becoming increasingly complex and dynamic, formal leaders may not be able to fulfill all leadership functions, making informal leadership an essential component of team success [1–3], there is growing interest in leadership patterns beyond the formal leadership role [4]. The ‘distributed leadership’ considers leadership as a practice pattern in which the responsibility and power are unlimited to exclusively one or a few formal leaders but are distributed between formal and informal leaders [5–8]. Harris had asserted that distributed leadership could imply a fundamental re-conceptualization of leadership as a practice and challenge conventional wisdom about the relationship between formal leadership and organizational performance [9]. The acknowledgment of such distributed leadership phenomena has fueled inquiry into the identification of leadership and the organizational consequences of individual behavior strategy [2,10,11].

Questions surrounding distributed leadership, while seemingly simple on the surface, have posed a complex challenge for researchers as they have worked to better understand the underlying mechanisms about leadership roles, responsibilities, activities and functions, which represents relational activities and processes of a team constituted and shaped by the interactions among team members and the team context [12]. Spillane et al. stated that distributed leadership is viewed as a pattern of the interactions among leaders, followers, and the situations in which they are [7]. Fairhurst and Cooren noted that leadership is constituted through the interactions of a plethora of actants, including followers [13]. According to Thorpe et al., distributed leadership alludes to various configurations that arise from the exercise of influence that produces interdependent and conjoint action [14]. Leadership roles, responsibilities, activities and functions are considered emergent properties and distributed in various ways throughout the team [12]. It is thoroughly recognized that the decision-making behavior of various people inside the team plays a highly significant role in promoting optimal cooperation and team performance. Nevertheless, little attention is paid to the investigation into the underlying mechanisms about how people in a team cooperate to initiate effective interactions and enhance team performance. More recently, scholars from a variety of disciplines have been applying evolutionary theory to help unify findings on leadership [15,16]. Accordingly, in most evolutionary behavioral models, decision-makers expand to maximize their performance but often encounter conflict with other decision-makers in a dynamic, unfolding, and complex organizational environment [17]. If, from an evolutionary perspective, the formal leader, the informal leader, and the ordinary employee are ‘relational beings’ who constitute and gamble with each other, is it possible to develop a steady, efficient distributed leadership pattern? In acknowledging this question, a tripartite evolutionary game model is needed.

Evolutionary game theory (EGT) was initially applied to biological conditions, in which the evolutionary phenomenon is described with the mathematical theory of games to explain animal conflicts and strategies [18,19]. Specifically, animals with high fitness have more evolutionary advantages. Moreover, when EGT is used to guide people’s decisions, it is shown that individuals with high payoffs have more evolutionary advantages [20–23]. Since the principle of EGT considers the strategy payoffs of both the player and the others, introducing EGT is a better understanding of the cost and benefit of the decision when facing the challenges of a complex and uncertain environment [24–27]. Evolutionary games can be used flexibly in two-player [28–30], three-player [31–33], or multi-player games [20–23,34].

In the distributed leadership pattern, the players are bounded rational and adjust their strategies dynamically by observing and comparing payoff with others [26,35]. Therefore, EGT is suitable for studying the long-term dynamic game in distributed leadership patterns. By linking formal leaders, informal leaders, and ordinary employees in teams and examining their effects on personal and team performance, this study extends the domain of distributed leadership patterns. From a distributed perspective, social interaction is a critical part of leadership practice. How formal leaders interact with others, how informal leaders interact with others, and how ordinary employees interact with formal and informal leaders, the reciprocal nature of distributed leadership is considered far more important than the precise leadership role or the particular leadership functions [9]. Therefore, this study constructs a tripartite evolutionary game model and analyzes the decision-making behavior of various actors in distributed leadership patterns. The study finds that the equilibrium of the three parties is affected by multiple factors. Analyzing and understanding patterns of influence get us much closer to the actual practice of distributed leadership.

Furthermore, we add depth to the literature on informal leadership by investigating the decision-making process of informal leadership [36]. The study of informal leadership in organization emerged with the discussion of interpersonal relationships between organizational members [1]. Informal leadership ‘occurs when team members who do not occupy formal leadership positions engage in influence behavior that helps their team determine shared goals, motivates task activity in pursuit of those goals, and creates a positive social climate’ [37]. The adaptive leadership theory proposes that the construction of informal leadership is determined by initiative leadership-emergence and leadership-empowering interactions among individuals [38]. We introduce the informal leaders into the evolutionary game model of distributed leadership, extending the field of informal leadership literature and, to some degree, the broader perspective of adaptive leadership theory.

Finally, we extend followership literature by examining the following behaviors of people acting in relation to formal or informal leaders. A current consensus is that the followership behavior of ordinary employees is a crucial component of the leadership process [39–42]. To escape the dilemma of the ‘incomplete theoretical construction’, it is critical to study followers as the center of the formal and informal leadership processes and how they enact followership [43]. By conducting a systematic theoretical exploration of the complex interaction among formal leadership, informal leadership, and ordinary employees, an economic model is proposed for the formalization of the previous idea, as well as interpersonal relationships and personal performance from a generally dynamic perspective. Unlike the previous research, our study does not address independent activities but rather the behavior of employees concerning a formal or informal leader. Overall, we propose a methodological innovation for comprehending how the followership behaviors of people act that influence the leadership behaviors.

The remainder of the paper is organized as follows. Section 2 conducts a detailed analysis of the decision-makers and proposes the assumptions of the tripartite evolutionary game model, which are the basis for subsequent analysis. Section 3 introduces evolutionary game analysis from the individual perspective, obtains the individual evolutionarily stable strategy (IESS), and analyzes the key parameters in the evolution process. The following Section 4 realizes the evolutionary analysis from the holistic perspective and obtains the ESS. The effect of parameter variations on the final evolution results is also analyzed and the correctness of the ESS is confirmed in the simulation section. Finally, Sections 5 and 6 provide discussions and conclusions.

## 2. Simple models

### 2.1. Model assumptions

Distributed leadership pattern distributes the requisite leadership functions among team members via a division of labor across time [8,38]. In the distributed leadership pattern, to analyze the behavior of decision-makers, we assume that there is one formal leader with a formal leadership position, one informal leader with heterogeneous knowledge, and one ordinary employee. These three decision-makers are ‘ bounded relational beings’ and constitute each other in an unfolding, dynamic relational context [40]. Based on the bounded rationality of these three decision-makers, we apply the evolutionary game to develop the analysis. For brevity, a subscript 1 in the lower right corner of a parameter means that this parameter corresponds to the formal leader. In contrast, 2 or 3 at the same position of a parameter means that this parameter is the corresponding parameter of the informal leader or ordinary employee.

In the evolutionary process, the equilibrium is realized gradually after the continuous strategy adjustments of all decision-makers, and the speed of strategy adjustments depends on the benefit of strategies. To achieve the subsequent evolution analysis, the following model assumptions are first made.

**Assumption 1** Since organizations have become increasingly knowledge-based with professional work and innovation, the formal leader should recognize that he/she does not necessarily have all the answers and seeks to encourage the strengths and contributions of others [44]. The formal leader may have ‘the vision’ to coordinate it while lacking the heterogeneous knowledge to decide on task-specific matters [11,45]. An active informal leader with heterogeneous knowledge may emerge to help the formal leader supervise the ordinary employees [2,46]. In addition, it is often stated that without followers, there can be no leaders [39,43,47]. The leadership influencing ordinary employees relies not only on a leader’s formal position or an informal leader’s abilities, but also on ordinary employee’s decisions on whether to follow or not [15,37,48]. Therefore, the strategy space of the formal leader is {empowering, non-empowering}, and the corresponding probabilities that the formal leader chooses {empowering} and {non-empowering} are set as *x* and 1−*x* respectively. The strategy spaces of the informal leader and the ordinary employee are {emerging, non-emerging} and {following, non-following}. Similarly, the corresponding probabilities of the four strategies are set as *y*, 1−*y*, *z*, and 1−*z* where *x*,*y*,*z*∈[0,1].

**Assumption 2** Team performance results from the combined effect of ordinary employee’s strategy and market mechanism, and team performance directly and positively affects the benefit of the formal leader [11,49,50]. Thus, it is assumed that when the ordinary employee adopts the {following} strategy to work actively, a benefit *R*_1*h*_ could be obtained for the formal leader, and correspondingly, a benefit *R*_1*l*_ is gained by the formal leader when the ordinary employee adopts {non-following}.

**Assumption 3** An active informal leader with heterogeneous knowledge may emerge to help the formal leader guide and supervise the ordinary employees [2,20–23,46]. Here we set that the informal leader with the {emerging} strategy will replace the formal leader with the {empowering} strategy to exercise supervision power on the ordinary employee. While some may welcome an opportunity for increased power, status, reputation, and performance [2,10,51,52], others may suffer from psychological distress, such as anxiety and fear of failure [42,52,53]. Therefore, the payment *P* from the formal leader to the informal leader is necessary. Then during the supervision of the informal leader, he/she could decide whether to shield the {non-following} behavior of the ordinary employee. Since whether to shield is not the key strategy to the main analysis, we simplify by setting the probability of his/her choosing to shield to *p* and the probability of not choosing to shield to 1−*p*. If the informal leader intends to shield, benefit *T* will be claimed from the ordinary employee, which could be manifested as some gifts. However, if the informal leader does not shield, the ordinary employee will lose the year-end bonus *B*. These two parameters should satisfy *B*>*T*. Specifically, based on the strategy combination {{empowering}, {emerging}, {non-following}} with the setting about *p*, the pay from the ordinary employee to the informal leader is *pT*, and the pay from the formal leader to the ordinary employee is *pB*.

**Assumption 4** When the formal leader chooses the {non-empowering} strategy or the informal leader chooses the {non-emerging} strategy, the formal leader needs to exercise supervision power by himself/herself with the cost *C*_1*s*_ [54]. In the process of the formal leader’s supervision, the ordinary employee with the {non-following} strategy will not only lose the year-end bonus *B*, but also pay a fine *F*. The formal leader has a further power distance from the ordinary employee, as the informal leader is closer to the ordinary employee [55], which is why the formal leader costs the transfer pay *P* and empowers an informal leader to supervise. Thus *C*_1*s*_>*P* is set.

**Assumption 5** In this model, the informal leader has a basic salary *R*_2_, and he/she could choose to take the {emerging} strategy to achieve the benefit *P* from the formal leader with {empowering}. In the case that the informal leader pays the cost *C*_2_ to choose {emerging}, the ordinary employee can gain *K* due to the knowledge spillover. Then if the informal leader with {emerging} encounters the formal leader with {empowering}, he/she will obtain the supervision power on the ordinary employee, which will produce a new cost *C*_2*s*_. The cost *C*_2*s*_ is the prerequisite for the implementation of the supervision. Therefore, the formal leader who chooses {empowering} should make efforts to ensure the informal leader who chooses {emerging} gets more benefit. Thus *P*>*C*_2_+*C*_2*s*_ is set.

**Assumption 6** The income of the ordinary employee can be divided into two parts: basic salary *R*_3_ and year-end bonus *B*. When the ordinary employee takes the {non-following} strategy, he/she will get extra benefit *E*. For example, the ordinary employee can obtain benefit through other channels during working hours, or simply use working hours for leisure to achieve individual satisfaction, which is harmful to team performance. In contrast, when the ordinary employee adopts the {following} strategy to work actively, he/she needs to pay an opportunity cost *C*_3_ to achieve his/her income. Therefore, to ensure the possibility of the ordinary employee adopting {following}, *B*>*C*_3_ is set.

In our tripartite evolutionary game model, we set the formal leader, the informal leader, and the ordinary employee as key decision-makers. Then as described in Assumption 1, the corresponding strategy spaces of these decision-makers are defined as {empowering, non-empowering}, {emerging, non-emerging} and {following, non-following}. Therefore, there are eight possible strategy combinations, for example, {{empowering}, {emerging}, {following}}. Based on any strategy combination, the benefit of any decision-maker can be obtained due to the above assumptions. To develop the subsequent analysis more clearly, we summarized all the parameters in the above assumptions and re-presented them in Table 1.

**Table 1:** Notations list including all key symbols.

Symbol	Description	Symbol	Description
*R*_1*h*_	The benefit of formal leader when ordinary employee adopts {following}	*R*_1*l*_	The benefit of formal leader when an ordinary employee adopts {non-following}
*R*_2_	The basic salary of informal leader	*R*_3_	The basic salary of ordinary employee
*C*_1*s*_	The supervision cost of formal leader	*C*_2*s*_	The supervision cost of informal leader
*C*_2_	The cost of informal leader with {emerging} strategy	*C*_3_	The cost of ordinary employee with {following} strategy
*P*	The payment of formal leader with {empowering} strategy to informal leader with {emerging} strategy	*F*	The fine paid by ordinary employee with {non-following} strategy to formal leader with supervision power
*p*	The probability of informal leader with supervision power choosing to shield ordinary employee	*T*	The benefit of informal leader with supervision power from ordinary employee with {non-following} strategy
*B*	The year-end bonus of ordinary employee	*E*	The extra benefit of ordinary employee with {non-following} strategy
*K*	The gain of ordinary employee from informal leader with {emerging} strategy		

### 2.2 Evolutionary game model

The strategy spaces of the three decision-makers, the formal leader, the informal leader, and the ordinary employee, are defined as {empowering, non-empowering}, {emerging, non-emerging} and {following, non-following}. The strategies of these three decision-makers will form a strategy combination. In this article, eight possible strategy combinations are shown in Table 2.

**Table 2:** Strategy matrix of tripartite evolutionary game.

		Informal leader	Ordinary employee	
			Following (*z*)	Non-following (1−*z*)
Formal leader	Empowering (*x*)	Emerging (*y*)	*X*_1_, *Y*_1_, *Z*_1_	*X*_2_, *Y*_2_, *Z*_2_
		Non-emerging (1−*y*)	*X*_3_, *Y*_3_, *Z*_3_	*X*_4_, *Y*_4_, *Z*_4_
	Non-empowering (1−*x*)	Emerging (*y*)	*X*_5_, *Y*_5_, *Z*_5_	*X*_6_, *Y*_6_, *Z*_6_
		Non-emerging (1−*y*)	*X*_7_, *Y*_7_, *Z*_7_	*X*_8_, *Y*_8_, *Z*_8_

In Table 2, we set that the formal leader, the informal leader, and the ordinary employee can obtain *X*_1_, *Y*_1_ and *Z*_1_ respectively with the strategy combination{{empowering}, {emerging}, {following}}. Similar settings are also made for the other seven strategy combinations. Then based on the strategy matrix in Table 2 and the six assumptions above, the benefit matrix in Table 3 can be constructed.

**Table 3:** Benefit matrix of tripartite evolutionary game.

		Informal leader	Ordinary employee	
			Following (*z*)	Non-following (1−*z*)
Formal leader	Empowering (*x*)	Emerging (*y*)	*R*_1*h*_−*B*−*P*	*R*_1*l*_−*pB*−*P*
			*R*_2_−*C*_2_−*C*_2*s*_+*P*	*R*_2_−*C*_2_−*C*_2*s*_+*P+pT*
			*R*_3_+*B*−*C*_3_+*K*	*R*_3_+*E*+*K*+*p*(*B*−*T*)
		Non-emerging (1−*y*)	*R*_1*h*_−*B*−*C*_1*s*_	*R*_1*l*_−*C*_1*s*_+*F*
			*R*_2_	*R*_2_
			*R*_3_+*B*−*C*_3_	*R*_3_+*E*−*F*
	Non-empowering (1−*x*)	Emerging (*y*)	*R*_1*h*_−*B*−*C*_1*s*_	*R*_1*l*_−*C*_1*s*_+*F*
			*R*_2_−*C*_2_	*R*_2_−*C*_2_
			*R*_3_+*B*−*C*_3_+*K*	*R*_3_+*E*−*F*+*K*
		Non-emerging (1−*y*)	*R*_1*h*_−*B*−*C*_1*s*_	*R*_1*l*_−*C*_1*s*_+*F*
			*R*_2_	*R*_2_
			*R*_3_+*B*−*C*_3_	*R*_3_+*E*−*F*

By comprehensively considering Tables 2 and 3, decision-makers corresponding benefits under different strategy combinations can be learned. For example, for the strategy combination {{empowering}, {emerging}, {following}}, the benefits of the three decision-makers are *X*_1_ = *R*_1*h*_−*B*−*P*, *Y*_1_ = *R*_2_−*C*_2_−*C*_2*s*_+*P* and *Z*_1_ = *R*_3_+*B*−*C*_3_+*K* respectively. A more detailed explanation of the benefits is shown below.

For the strategy combination {{empowering}, {emerging}, {following}}, we set the ordinary employee with the {following} strategy as the direct cause of team performance, and the income of the formal leader with the {empowering} strategy is positively related to the team performance, so the formal leader obtains the income *R*_1*h*_. Then for the informal leader with the {emerging} strategy, he/she ensures a stable income *R*_2_ with the fixed cost C_2_, and pays the cost *C*_2*s*_ due to the existence of informal leader’s supervision. Same for the ordinary employee with {following} strategy, he/she also ensures the fixed income *R*_3_ and the cost *C*_3_, and obtains the benefit *K* from the {emerging} strategy chosen by the informal leader. In addition to the above seven parameters, there are also two parameters representing payments: *B* and *P*. *B* is the payment from the formal leader with {empowering} to the ordinary employee with {following}, and *P* is the payment from the formal leader with {empowering} to the informal leader with {emerging}.

## 3. Individual evolutionarily stable strategy analysis

In the distributed leadership pattern, the decision-makers are bounded rational and adjust their strategies dynamically by observing and comparing payoff with others [26,35]. Therefore, EGT is suitable for studying the long-term dynamic game of bounded rational players in distributed leadership patterns. In EGT, the replicator dynamic equation [26,35] is set as

$$\frac{dq}{dt}=q(1-q)(E_{q}-E_{1-q}),$$ (1)

where *q* and 1−*q* represent the probabilities of two arbitrary possible strategies, while *E*_*q*_ and *E*_1−*q*_ are the expected benefits of these two strategies. Therefore, the evolutionary speed $\frac{dq}{dt}$ of the probability *q* over time *t* depends on the contact rate *q*(1−*q*) of two strategies and the evolution advantage *E*_*q*_−*E*_1−*q*_ of *q* over 1−*q*.

### 3.1 Strategy analysis of formal leader

Based on Table 2, the expected benefit of the formal leader with {empowering} strategy can be calculated as $E_{x}=yzX_{1}+y(1-z)X_{2}+(1-y)zX_{3}+(1-y)(1-z)X_{4}$. Then if the formal leader chooses {non-empowering}, the expected benefit will transform into $E_{1-x}=yzX_{5}+y(1-z)X_{6}+(1-y)zX_{7}+(1-y)(1-z)X_{8}$.

According to Eq (1), the replicator dynamic equation of formal leader is $F(x)=\frac{dx}{dt}=x(1-x)(E_{x}-E_{1-x})$, and Eq (2) can be calculated based on Table 3.

$$F(x)=\frac{dx}{dt}=x(1-x)(E_{x}-E_{1-x})=x(1-x)yg(z),$$ (2)

where *g*(*z*) = *C*_1*s*_−*P*−(1−*z*)(*F*+*pB*).

The condition for achieving equilibrium in the evolutionary game is $F(x)=\frac{dx}{dt}=0$, which means that the probability of choosing {empowering} strategy *x* does not change with time *t*. However, it is not enough to gain the individual evolutionary stable strategy (IESS), which requires the equilibrium to remain stable under external interference. For the clarity of explanation, *x* at the equilibrium point is set as x*.

To ensure the x* is an IESS, it is required that x* is still an equilibrium point after being disturbed by external factors, meaning there should be a positive number *δ*, so that $F(x)=\frac{dx}{dt}>0$ while *x* is in the range of (x*−*δ*, x*), which means that *x* will increase over time *t* until it reaches x*. In addition, it also satisfies $F(x)=\frac{dx}{dt}<0$ while *x* is in the range of (x*, x*+*δ*), where *x* will decrease over time *t* until it reaches x*. Therefore, if *x* deviates from the point x* due to external factors and is in the neighborhood of x*, *x* will still return to the original equilibrium point x* over time *t*. Thus, in the *x* dimension, there must be a positive number *δ*′ and *δ*′≤*δ*, so that *F*(*x*) decreases monotonically with the *x* in the range of (x*−*δ*′, x*+*δ*′). In other words, $\frac{dF(x)}{dx}<0$ while *x*∈(x*−*δ*′, x*+*δ*′). Hence, Proposition 1 is obtained.

**Proposition 1** The conditions for the tripartite evolutionary game to achieve IESS in the *x* dimension are $F(x)=\frac{dx}{dt}=0$ and $\frac{dF(x)}{dx}<0$.

Combined with the expression of *F*(*x*) in Eq (2), we can see that the formal leader who intends to achieve IESS needs to satisfy

$$\left\{ \begin{array}{l} F(x)=x(1-x)yg(z)=0\\ \frac{dF(x)}{dx}=(1-2x)yg(z)<0 \end{array}.\right.$$ (3)

Because of *g*(*z*) = [*C*_1*s*_−*P*−(1−*z*)(*F*+*pB*)] and $\frac{dg(z)}{dz}=F+pB>0$, two solutions can be obtained by solving Eq (3). The first one is *x* = 0, *y*≠0 and *z*<z*, where z* satisfies *g*(z*) = 0 and $z^{*}=\frac{-C_{1s}+P}{F+pB}+1$, and the second is *x* = 1, *y*≠0 and *z*>z*. Thus, Proposition 2 can be obtained by analyzing the two solutions.

**Proposition 2** If *y*≠0 and $z<z^{*}=\frac{-C_{1s}+P}{F+pB}+1$, the IESS of the formal leader is *x* = 0. And if *y*≠0 and $z>z^{*}=\frac{-C_{1s}+P}{F+pB}+1$, the IESS is *x* = 1. However, if *y* = 0 or $z=z^{*}=\frac{-C_{1s}+P}{F+pB}+1$, there will be no stable equilibrium point.

**Fig 1:**
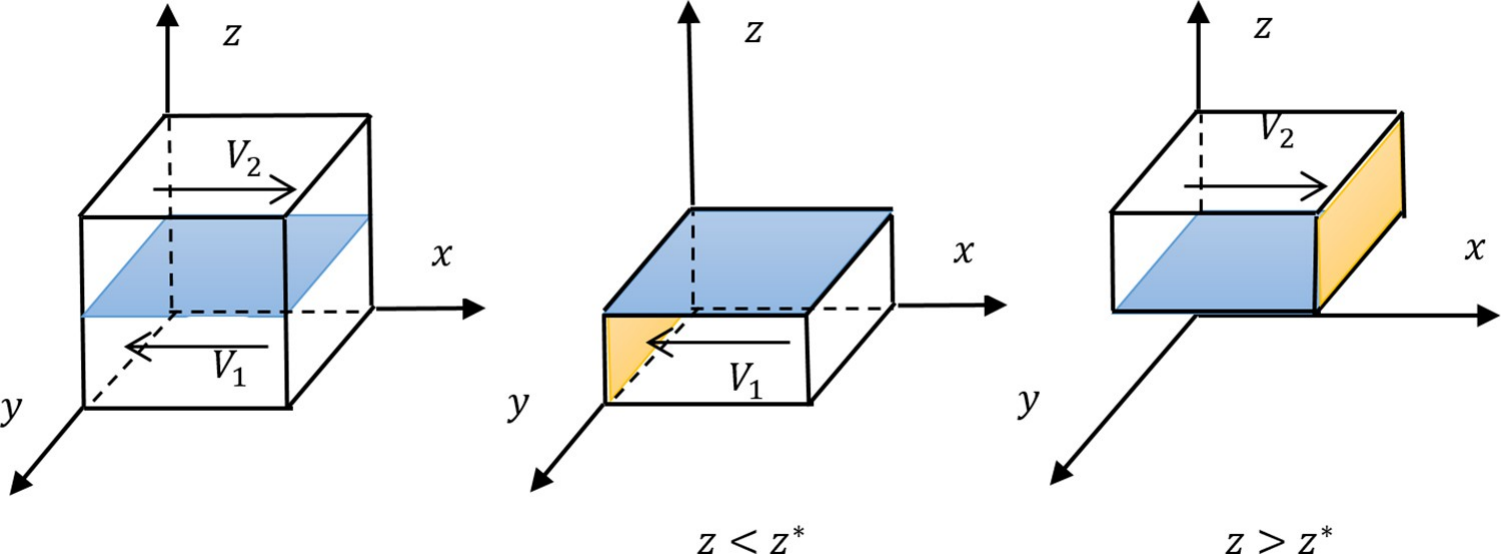
The evolution trend diagram of the formal leader.

Note that *C*_1*s*_>*P* in Assumption 4, so $z^{*}=\frac{-C_{1s}+P}{F+pB}+1<1$. If z*∈[0,1), Proposition 2 can be presented as the evolution trend diagram of Fig 1. And if z*<0, we set the volume *V*_1_ in Fig 1 to be zero.

The pure strategy space of the formal leader is {empowering, non-empowering}. When the formal leader decides to take {empowering} strategy and {non-empowering} strategy with probabilities *x* and 1−*x* respectively, the mixed strategy space of formal leader is produced as {*x*|*x*∈[0, 1]}. The formal leader, the informal leader, and the ordinary employee all can select a mixed strategy from their own mixed strategy space, which can form a mixed strategy combination, and then the whole of the possible mixed strategy combination is represented as a cube in the leftmost subfigure in Fig 1.

As shown in the leftmost subfigure of Fig 1, all the mixed strategy combinations are divided into two parts by the plane *z* = z*, named *V*_1_ and *V*_2_ respectively. *V*_1_ conforms to the setting of *y*≠0 and *z*<z*, and the formal leader’s IESS is *x* = 0 based on Proposition 2, so there is an arrow opposite to the direction of the *x* axis. The *V*_1_ is presented alone in the middle of Fig 1, and the surface formed by *x* = 0 is marked in orange. *V*_2_ conforms to the setting of *y*≠0 and *z*>z*, the formal leader’s IESS is *x* = 1, so there is an arrow in the same direction as the *x* axis, shown in the rightmost subfigure, and the surface formed by *x* = 1 is marked in orange.

As z* increases, the plane *z* = z* in Fig 1 will increase, and the volume of *V*_2_ will decrease, so the possibility of choosing {empowering} strategy by the formal leader will decrease accordingly. While if z* decreases, the plane will decrease, the volume of *V*_2_ will increase, and the formal leader will choose a great probability *x* to implement {empowering}. The value of z* is negatively related to the probability *x*.

Since $z^{*}=\frac{-C_{1s}+P}{F+pB}+1$, Proposition 3 can be obtained by analyzing the related parameters of z*. For example, z* is negatively related to *x* and z* is also negatively related to *C*_1*s*_. Thus, the correlation between *x* and *C*_1*s*_ is positive.

**Proposition 3** The probability of the formal leader taking {empowering} strategy *x* is positively correlated with the parameter *C*_1*s*_, and is negatively correlated with the parameters *P*, *p*, *B*, and *F*. The probability *x* is also positively correlated with the probability of the ordinary employee taking {following} strategy *z*.

The parameter *C*_1*s*_ is the supervision cost paid by the formal leader when he/she supervises the ordinary employee. The increase of the supervision cost will impel the formal leader to prefer the {empowering} strategy and encourage the informal leader to implement supervision, so that the probability *x* will increase accordingly. The parameters *P*, *p*, and *B* are all positively related to the payment of the formal leader who chooses {empowering}. Specifically, the parameter *P* is the payment from the formal leader to the informal leader when the informal leader supervises the ordinary employee. The combination of the parameters *p* and *B* reflects the expected bonus that the formal leader also pays. Therefore, the increase in the three parameters will prompt the formal leader not to choose {empowering}. The final *F* is the penalty that the formal leader with the {non-empowering} strategy obtained from the ordinary employee with the {non-following} strategy. The increase of *F* is more beneficial to the formal leader with the {non-empowering} strategy. Therefore, the parameter *F* is also negatively related to the probability *x*.

Finally, a supplementary explanation is made of the relationship between probability *x* and probability *z*. In Fig 1, in the case that the probability *z* is significant and exceeds z*, the formal leader is more willing to take {empowering} strategy, which means that when the ordinary employee adopts {following} strategy and actively works, the formal leader is more willing to empower the informal leader to supervise the ordinary employee, which is more effective.

### 3.2 Evolution analysis of the informal leader

Using the same analytical frame as the analysis of the formal leader, the expected benefit of the informal leader with {emerging} strategy is $E_{y}=xzY_{1}+x(1-z)Y_{2}+(1-x)zY_{5}+(1-x)(1-z)Y_{6}$, while the informal leader choosing {non-emerging} can obtain $E_{1-y}=xzY_{3}+x(1-z)Y_{4}+(1-x)zY_{7}+(1-x)(1-z)Y_{8}$. Based on Table 3, the replicator dynamic equation of the informal leader can be constructed, which is shown as Eq (4).

$$F(y)=\frac{dy}{dt}=y(1-y)(E_{y}-E_{1-y})=y(1-y)h(x,z),$$ (4)

where $h(x,z)=x(P+pT-C_{2s})-C_{2}-xzpT$. Similar to Proposition 1, the conditions of realization of IESS in the *y* dimension are *F*(*y*) = 0 and $\frac{dF(y)}{dy}<0$. By combining with Eq (4), we can see that the IESS of the informal leader is obtained by solving

$$\left\{ \begin{array}{l} F(y)=y(1-y)h(x,z)=0\\ \frac{dF(y)}{dy}=(1-2y)h(x,z)<0 \end{array}.\right.$$ (5)

Since *P*>*C*_2*s*_+*C*_2_ that shown in Assumption 5, we have $\frac{dh(x,z)}{dx}=P+(1-z)pT-C_{2s}>0$. Two solutions can be gained by solving Eq (5). The first solution is *y* = 0 and *x*<x*, and the second is *y* = 1 and *x*>x*, where $x^{*}=\frac{C_{2}}{P+(1-z)pT-C_{2s}}$. Thus, we obtain the following proposition.

**Proposition 4** If $x<x^{*}=\frac{C_{2}}{P+(1-z)pT-C_{2s}}$, the IESS of the informal leader is *y* = 0, and if $x>x^{*}=\frac{C_{2}}{P+(1-z)pT-C_{2s}}$, the IESS of the informal leader is *y* = 1.

**Fig 2:**
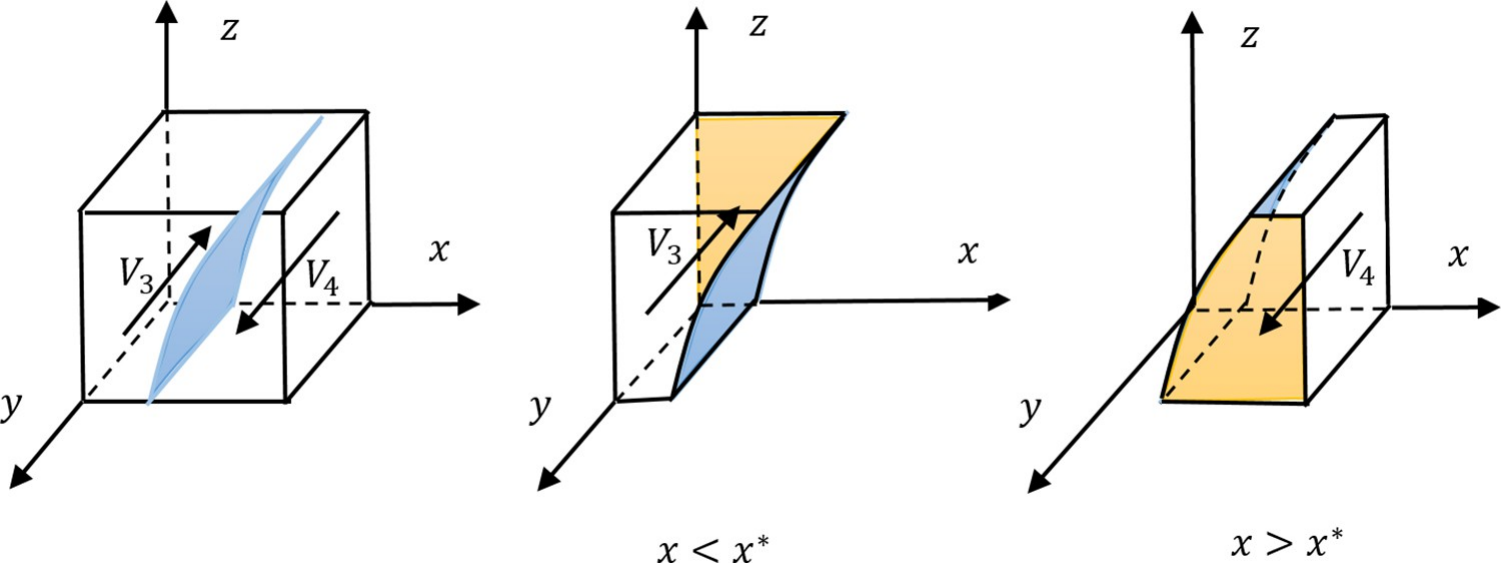
The evolution trend diagram of the informal leader.

Since *P*>*C*_2*s*_+*C*_2_ is given by Assumption 5, it is known that $x^{*}=\frac{C_{2}}{P+(1-z)pT-C_{2s}}\in (0,1)$. According to Proposition 4, the evolution trend diagram can be formed in Fig 2.

In the leftmost subfigure of Fig 2, all the mixed strategy combinations are divided into two parts, *V*_3_ and *V*_4_, with the plane *x* = x*. *V*_3_ meets the condition of *x*<x*, and the volume of *V*_3_ represents the probability that the informal leader chooses {non-emerging} while the IESS is *y* = 0. *V*_4_ conforms to *x*>x*, and the volume of *V*_4_ represents the probability of informal leader’s choice of {emerging} where the IESS is *y* = 1. According to the IESSs in *V*_3_ and *V*_4_, two arrows representing the evolution trend are drawn, and the surfaces formed by the IESSs are drawn in orange.

When the plane *x* = x* moves to the right resulting from the change of parameter, the volume of *V*_4_ decreases and the possibility of the informal leader’s choice of {emerging} decreases too, while the plane *x* = x* moves to the left, the volume of *V*_4_ will decrease and the informal leader is more likely to choose {emerging} accordingly. Therefore, the value of x* is negatively related to the probability *y*. Since $x^{*}=\frac{C_{2}}{P+(1-z)pT-C_{2s}}$, Proposition 5 can be obtained by combining the correlations between x* and each parameter.

**Proposition 5** The probability *y* is positively correlated with parameters *P*, *p*, and *T*, and negatively correlated with parameters *C*_2_ and *C*_2*s*_. Moreover, the probability *y* is positively correlated with the probability *x*, and negatively correlated with the probability *z*.

The parameters *P*, *p*, and *T* affect the benefit of the informal leader who takes the {emerging} strategy. Concretely, the parameter *P* is the benefit of the informal leader with {emerging} obtained from the formal leader with {empowering}, and the combination of *p* and *T* means the benefit of the informal leader with {emerging} obtained from the ordinary employee with {non-following}. The increase in the three parameters will inevitably prompt the informal leader to adopt the {emerging} strategy. The parameters *C*_2_ as the emerging cost and *C*_2*s*_ as the interpersonal risk cost are paid by the informal leader with {emerging} in a specific situation, and the increase of either of them is not conducive to adopting {emerging} for the informal leader.

The formal leader with {empowering} provides the informal leader an opportunity to implement supervision and achieve high returns. Suppose the formal leader increases the probability *x*, the informal leader will also increase probability *y* correspondingly, and the ordinary employee with {non-following} strategy should pay to seek a shield from the informal leader with regulatory power, which is not conducive to the ordinary employee. Thus, the probability *y* and probability *z* are negatively correlated.

### 3.3 Strategy analysis of ordinary employee

The expected benefit of the ordinary employee who chooses the {following} strategy is $E_{z}=xyZ_{1}+x(1-y)Z_{3}+(1-x)yZ_{5}+(1-x)(1-y)Z_{7}$, and the expected benefit of the ordinary employee who chooses {non-following} is $E_{1-z}=xyZ_{2}+x(1-y)Z_{4}+(1-x)yZ_{6}+(1-x)(1-y)Z_{8}$. Thus, based on Table 3, the replicator dynamic Eq (6) of the ordinary employee can be constructed.

$$F(z)=\frac{dz}{dt}=z(1-z)(E_{z}-E_{1-z})=z(1-z)l(x,y),$$ (6)

where $l(x,y)=B+F-E-C_{3}-xy(F+pB-pT)$. Similar to Proposition 1, it can be obtained that the conditions for the TEG to achieve IESS in the *z* dimension are F(z)=dzdt=0 and dF(z)dz<0.

Combining with Eq (6), it can be inferred that the realization of the IESS needs to meet

$$\left\{ \begin{array}{l} F(z)=z(1-z)l(x,y)=0\\ \frac{dF(z)}{dz}=(1-2z)l(x,y)<0 \end{array}.\right.$$ (7)

Because of Assumption 3, it follows that *B*>*T*, so $\frac{dl(x,y)}{dy}=-x(F+pB-pT)<0$. Two solutions can be gained by solving Eq (7). The first solution is *z* = 1 and *y*<y*, and the second is *z* = 0 and *y*>y*, where $y^{*}=\frac{B+F-E-C_{3}}{x(F+pB-pT)}$. Thus, we obtain Proposition 6.

**Proposition 6** When $y<y^{*}=\frac{B+F-E-C_{3}}{x(F+pB-pT)}$, the IESS of ordinary employee is *z* = 1, and when $y>y^{*}=\frac{B+F-E-C_{3}}{x(F+pB-pT)}$, the IESS of an ordinary employee is *z* = 0.

**Fig 3:**
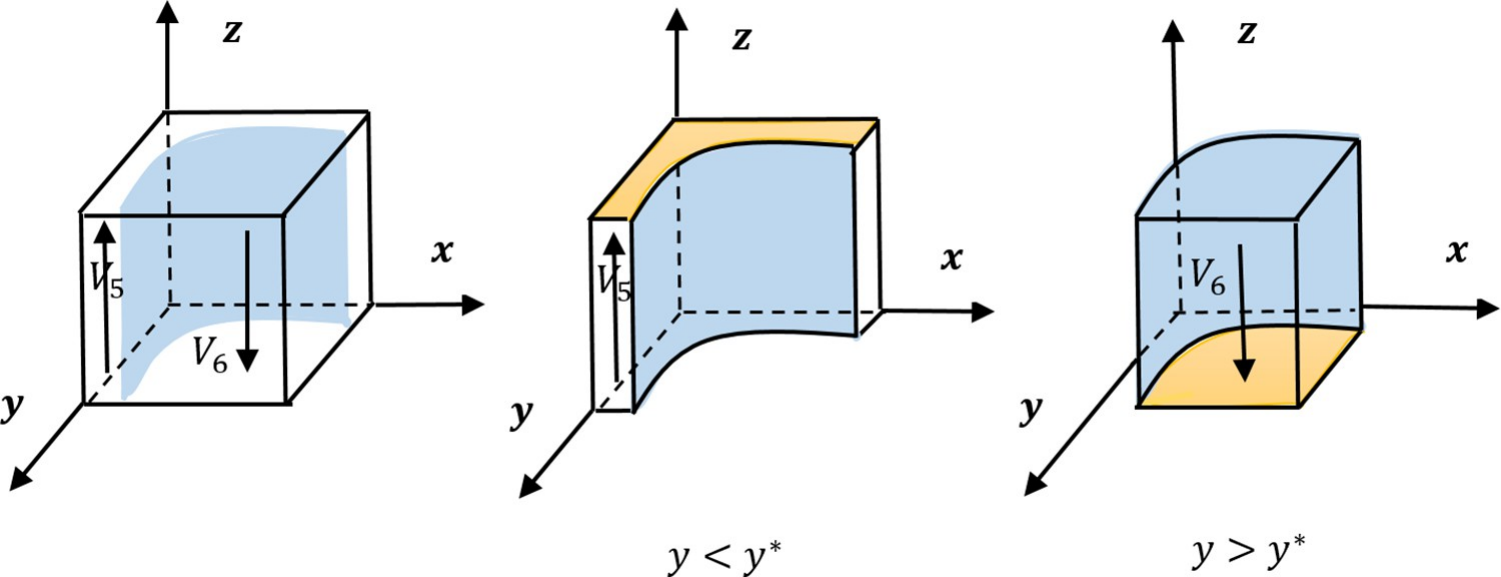
The evolution trend diagram of the ordinary employee.

If y*∈[0, 1], we can draw the evolution trend diagram in Fig 3. If y*<0, the only thing that should do is to set the volume of *V*_5_ in Fig 3 to 0. Similarly, if y*>1, the volume of *V*_6_ is set to 0.

The leftmost subfigure of Fig 3 divides the mixed strategy combination into two parts, *V*_5_ and *V*_6_, which correspond to the preconditions of *y*<y* and *y*>y* respectively. The division plane is *y* = y*. In *V*_5_ and *V*_6_, the IESSs of the ordinary employee are {following} and {non-following}, respectively. The surfaces represented by the IESSs in the two subfigures also are drawn in orange.

When the plane *y* = y* moves along the negative direction of the *y* axis, the volume of *V*_5_ decreases and the probability of forming *z* = 1 gets smaller. Moreover, the plane *y* = y* moves along the positive direction of the *y* axis, the volume of *V*_5_ increases and the probability of forming *z* = 1 will be greater. Therefore, the value of y* is positively correlated with the probability *z*. Since $y^{*}=\frac{B+F-E-C_{3}}{x(F+pB-pT)}$, Proposition 7 can be obtained by combining the correlations between y* and each parameter.

**Proposition 7** The probability *z* is positively correlated with parameter *T* and negatively correlated with parameters *E*, *C*_3_, and *p*. Moreover, the probability *z* is negatively correlated with the probability *x* and is negatively correlated with the probability *y*.

*T* represents the possible expenditures of the ordinary employee who takes the {non-following} strategy to the informal leader with supervision power for a shield. The increase of this parameter will prompt the ordinary employee to choose {following}. The parameter *E* is the benefit obtained by the ordinary employee by choosing {non-following}, the parameter *C*_3_ represents the cost of the ordinary employee’s choice of {following}, and the parameter *p* is the possibility that the ordinary employee with {non-following} are shielded. The increase in these three factors will directly cause the ordinary employee to prefer the {non-following} strategy.

It is known that when the probability of the formal leader adopting {empowering} *x* is large, the probability of the informal leader adopting {emerging} strategy *y* is also large, the informal leader’s supervision will become legitimate. The ordinary employee with {non-following} could be shielded by the informal leader, and thus the ordinary employee is willing to reduce the probability *z*.

## 4. Tripartite evolution stable strategy analysis

Section 3 analyzed the strategy evolution from three individual dimensions and obtained three IESSs. If these three decision-makers reach their respective IESSs simultaneously, the formed strategy combination is called ESS, theoretically realized by finding the intersection of the orange parts in Figs 1–3. However, the three figures are only schematic diagrams, and the analysis obtained based on them is not accurate enough. Therefore, the first method of Lyapunov [56] is adopted to obtain the ESS.

### 4.1 Conditional evolution stable strategy

Replicator dynamic equation determines the evolution speed of strategy over time. When the value of the replicator dynamic equation is equal to 0, it means the strategy will no longer change, and the equilibrium is reached. Thus, the simultaneous Eq (8) can be obtained by assigning all Eqs (2), (4) and (6) to 0.


$$\left\{ \begin{array}{c} F(x)=\frac{dx}{dt}=x(1-x)y(C_{1s}-P-(1-z)(F+pB))=0\\ F(y)=\frac{dy}{dt}=y(1-y)(-C_{2}+x(P+pT-C_{2s})-xzpT)=0\\ F(z)=\frac{dz}{dt}=z(1-z)(B+F-E-C_{3}-xy(F+pB-pT))=0 \end{array}.\right.$$ (8)

The values of *x*, *y* and *z* can be obtained by solving Eq (8). If the obtained values are all in the range [0, 1], the value combination is deemed to be a feasible solution, which is also called equilibrium. Since it is known that *B*>*T*, *C*_1*s*_>*P*, *P*>*C*_2_+*C*_2*s*_, and *B*>*C*_3_ under Assumptions 3, 4, 5, and 6, 13 equilibria can be obtained by solving Eq (8), of which 6 unconditional equilibria are *E*_1_{0, 1, 0}, *E*_2_{1, 1, 0}, *E*_3_{0, 1, 1}, *E*_4_{1, 1, 1}, *E*_5_{*x*_5_, 0, 0}, and *E*_6_{*x*_6_, 0, 1}, and the remaining 7 equilibria are conditional equilibria. They are presented in Table 4.

**Table 4:** Conditional equilibria of the tripartite evolutionary game.

Equilibrium	Conditions of equilibrium
*E*_7_{0, 1, z_7_}	*B*+*F*−*E*−*C*_3_ = 0
*E*_8_{*x*_8_, 0, z_8_}	*B*+*F*−*E*−*C*_3_ = 0
*E*_9_{1, 1, z_9_}	*B*−*E*−*C*_3_−(*pB*−*pT*) = 0
*E*_10_{*x*_10_, 1, 0}	*C*_1*s*_−*P*−*F*−*pB* = 0
*E*_11_{*a*_11_, *y*_11_, 0}	*C*_1*s*_−*P*−*F*−*pB* = 0
*E*_12_{*a*_12_, 1, *c*_12_}	*F*+*pB*+*P*−*C*_1*s*_>0, 0<*a*_12_<1
*E*_13_(*a*_13_, *b*_13_, *c*_13_}	*F*+*pB*+*P*−*C*_1*s*_>0, 0<*b*_13_<1

In Table 4, *x*, *y*, *z* with subscript represent variables, which can be any value in the interval [0,1], while *a*, b, c with subscript represent constants, specifically

$$a_{11}=\frac{C_{2}}{P+pT-C_{2s}},\;a_{12}=\frac{B+F-E-C_{3}}{F+pB-pT},\;c_{12}=c_{13}=\frac{F+pB+P-C_{1s}}{F+pB},$$

$$a_{13}=\frac{(F+pB)C_{2}}{\left(\left(P-C_{2s})B+T(C_{1s}-P\right))p+F(P-C_{2s}\right)},\;b_{13}=\frac{\left(\left(\left(P-C_{2s})B+T(C_{1s}-P\right))p+F(P-C_{2s}\right))(B+F-E-C_{3}\right)}{(F+pB)((B-T)p+F)C_{2}}.$$

The conditional equilibria can be written as the equilibria with new subscripts, where the conditions are placed. For example, the 7^th^ equilibrium is written as $E_{7}\;\{0,\;1,\;z_{7}\}{|}_{B+F-E-C_{3}=0}$, which can be simplified as *E*_7_{0, 1, z_7_} or *E*_7_.

To judge the stability of the 13 equilibria, the first method of Lyapunov is applied to convert the replicator dynamic Eqs in (8) into linear differential equations. The specific operation is made for obtaining (9) based on (8).


$$\left\{ \begin{array}{l} F(x)=\frac{dx}{dt}\approx \frac{\partial F(x)}{\partial x}x+\frac{\partial F(x)}{\partial y}y+\frac{\partial F(x)}{\partial z}z\\ F(y)=\frac{dy}{dt}\approx \frac{\partial F(y)}{\partial x}x+\frac{\partial F(y)}{\partial y}y+\frac{\partial F(y)}{\partial z}z\\ F(z)=\frac{dz}{dt}\approx \frac{\partial F(z)}{\partial x}x+\frac{\partial F(z)}{\partial y}y+\frac{\partial F(z)}{\partial z}z \end{array}\right..$$ (9)

The Jacobian Matrix (10) can be constructed by combining the coefficients of *x*, y *z* in (9). For example, since $\frac{\partial F(x)}{\partial x}=(1-2x)yg(Z)$, $\frac{\partial F(x)}{\partial y}=x(1-x)g(Z)$ and $\frac{\partial F(x)}{\partial z}=x(1-x)y(F+pB)$, the values on the right side of these three equations are put into the first row of the Jacobian Matrix (10).


$$J=\left[\begin{array}{ccc} \frac{\partial F(x)}{\partial x} & \frac{\partial F(x)}{\partial y} & \frac{\partial F(x)}{\partial z}\\ \frac{\partial F(y)}{\partial x} & \frac{\partial F(y)}{\partial y} & \frac{\partial F(y)}{\partial z}\\ \frac{\partial F(z)}{\partial x} & \frac{\partial F(z)}{\partial y} & \frac{\partial F(z)}{\partial z} \end{array}\right]=[\begin{array}{ccc} \left(1-2x)yg(z\right) & x(1-x)g(z) & x(1-x)y(F+pB)\\ y(1-y)((P+pT-C_{2s})-zpT) & \left(1-2y)h(x,z\right) & y(1-y)(-xpT)\\ z(1-z)(-y(F+pB-pT)) & z(1-z)(-x(F+pB-pT)) & \left(1-2z)l(x,y\right) \end{array}].$$ (10)

**Fig 4:**
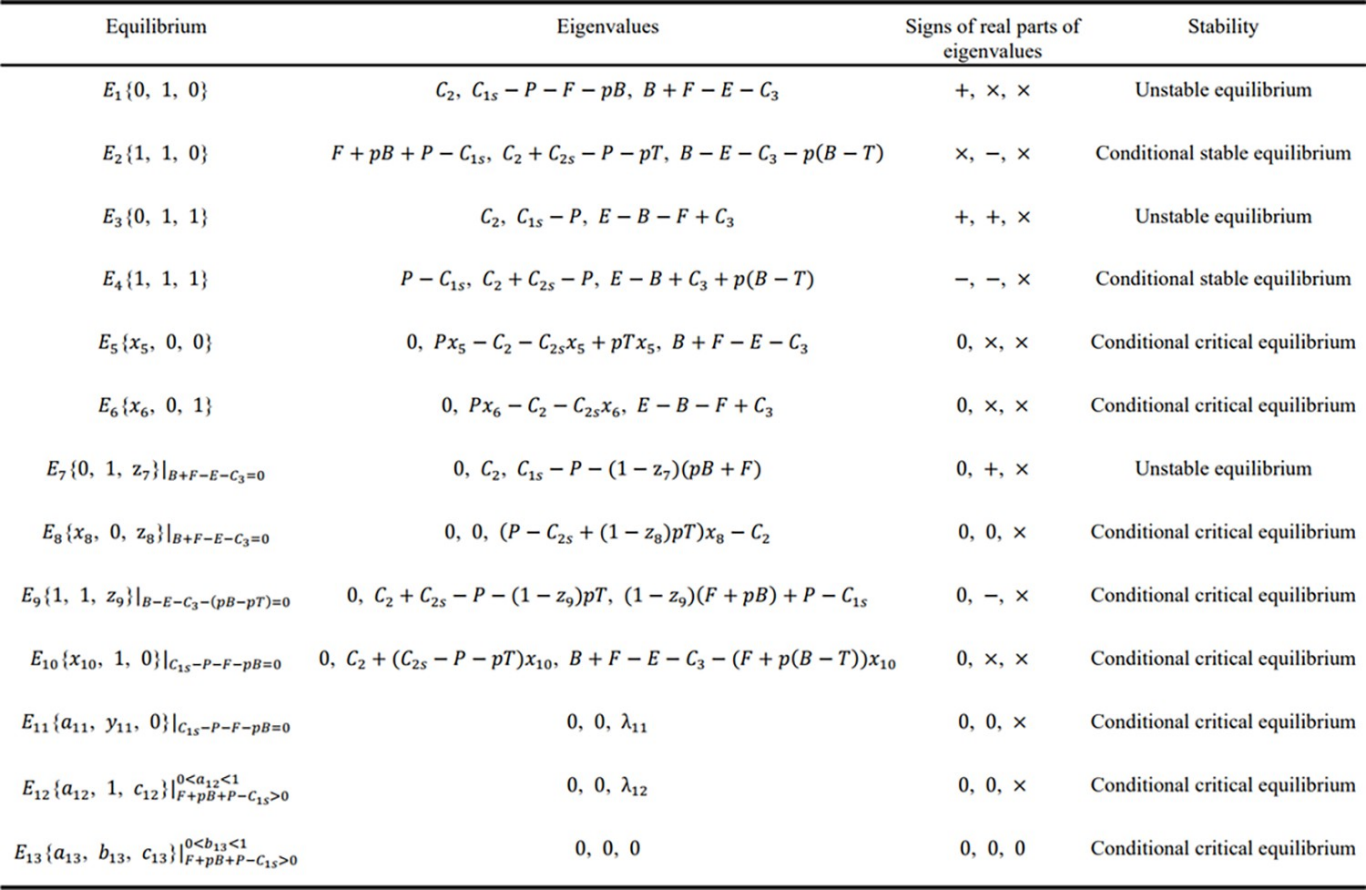
Stability analysis of equilibrium.

For each equilibrium with certain *x*, y, and *z*, three eigenvalues can be obtained for the corresponding Jacobian matrix. Relied on the first method of Lyapunov, if the real parts of the eigenvalues are all negative, the equilibrium is a stable equilibrium, which is also an ESS. While if some of the real parts of the eigenvalues are negative, and the others are 0, the equilibrium is critical. Finally, if any eigenvalue here has a positive real part, the corresponding equilibrium is unstable. The 13 equilibria are brought into Eq (10), and the corresponding eigenvalues can be obtained. The stabilities of the equilibria can be judged based on the signs of the real parts of the three eigenvalues, with the results summarized in Fig 4.

Fig 4 contains four possible results when judging the sign of the eigenvalue’s real part, which could be ‘0’, ‘+’, ‘−’, or ‘×’. The signs ‘0’, ‘+’, and ‘−’ indicate that the real part is 0, positive, and negative, respectively, and the sign ‘×’ means that the sign of the real part cannot be judged by using the existing conditions. However, the ‘×’ can be ‘−’ when it meets certain conditions. Therefore, the stable equilibrium that needs to meet specific conditions is called conditional stable equilibrium, and the critical equilibrium with specific conditions is called conditional critical equilibrium. Fig 4 shows that the conditional stable equilibria are only *E*_2_{1, 1, 0} and *E*_4_{1, 1, 1}, which are also called conditional evolutionarily stable strategies (CESS). We will analyze the conditions of the two CESSs in the next subsection, and we will realize the effective supervision of the distributed leadership pattern.

### 4.2 Evolutionary analysis and simulation

This article analyzes the ESS of the distributed leadership pattern, and the optimal ESS is the stable equilibrium where the ordinary employee adopts the {following} strategy and works hard, which is beneficial to the team performance. So, for the team performance, it is necessary to achieve *E*_4_{1, 1, 1} instead of *E*_2_{1, 1, 0}. We note that *E*_2_ must be an unstable equilibrium when *E*_4_ is an ESS because the signs of the real parts of the third eigenvalues are just opposite in Fig 4. Therefore, the goal is to make *E*_4_ an ESS.

The CESS *E*_4_{1, 1, 1} is an ESS when the condition *E*−*B*+*C*_3_+*p*(*B*−*T*)<0 is satisfied because then the real parts of the eigenvalues of the Jacobian Matrix all are negative.

Combining with Table 3, we see that *E*−*B*+*C*_3_+*p*(*B*−*T*) = *Z*_2_−*Z*_1_, thus the condition of the realization for *E*_4_ being ESS is only *Z*_2_<*Z*_1_. *E*_4_{1, 1, 1} with *x* = *y* = 1 indicates that the informal leader replaces the formal leader to exercise supervision power. Then *Z*_1_ is the benefit of the ordinary employee with the {following} strategy, and *Z*_2_ is the benefit of the ordinary employee with the {non-following} strategy. To ensure *Z*_2_<*Z*_1_ and the ordinary employee with the {following} strategy to obtain a higher benefit, the values of parameters *E*, *B*, *C*_3_, *p*, and *T* need to be reasonable, which are shown in Proposition 8.

**Proposition 8** When the informal leader replaces the formal leader to exercise supervision power, parameters *B* and *T* need to be appropriately increased and parameters *E*, *C*_3_ and *p* need to be appropriately decreased to ensure the ordinary employee takes the {following} strategy.

Parameters *B* and *C*_3_ represent the benefit and the cost of the ordinary employee with the {following} strategy. Increasing benefit *B* and reducing cost *C*_3_ will prompt the ordinary employee to actively choose the {following}. Whereas the parameters *T* and *p* represent the payment and the possibility of being shielded for the ordinary employee with the {non-following} strategy, and increasing the cost *T* and reducing the possibility *p* will make the ordinary employee abandon the {non-following} strategy and choose the {following} strategy. Finally, the parameter *E* is the extra benefit obtained by the ordinary employee taking the {non-following} strategy, and the decrease of *E* will inevitably cause the ordinary employee to adopt the {following}.

**Fig 5:**
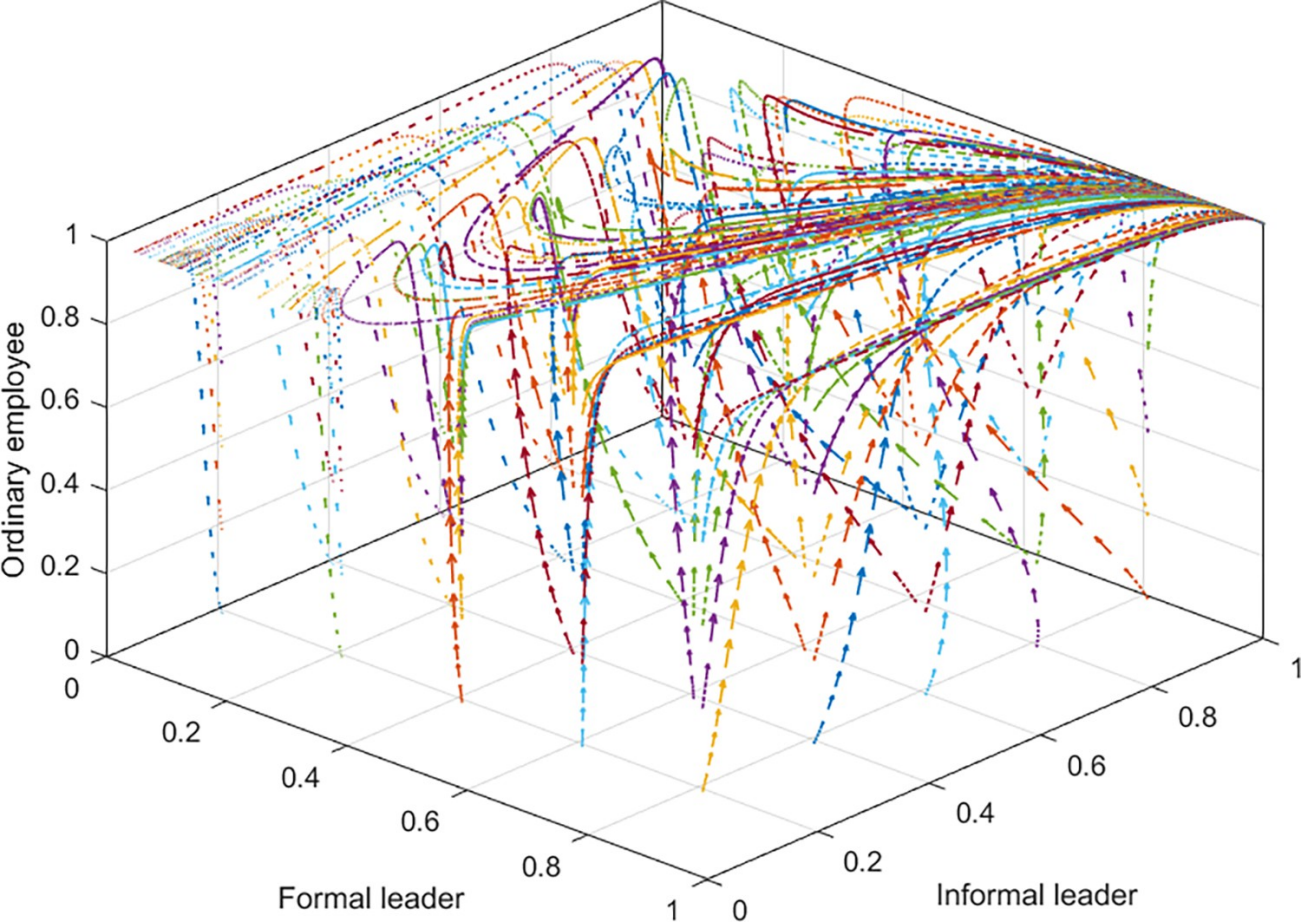
Simulation diagram of the tripartite evolutionary game under certain conditions.

It has been known that the condition for the CESS *E*_4_ to be an ESS is *E*−*B*+*C*_3_+*p*(*B*−*T*)<0, and the limitations for parameters are *B*>*T*, *C*_1*s*_>*P*, *P*>*C*_2_+*C*_2*s*_, and *B*>*C*_3_, which are shown in Assumptions 3, 4, 5, and 6. Thus we simply set that *C*_1*s*_ = 200, *P* = 180, *F* = 150, *p* = 0.5, *B* = 250, *C*_2_ = 50, *T* = 70, *C*_2*s*_ = 50, *E* = 30, *C*_3_ = 50. The evolution process is simulated and shown in Fig 5.

There are 125 solid lines with evolution directions in Fig 5, representing the evolution of 125 strategy combinations over time. The initial strategy combinations are evenly distributed in the cube, and the result is that they all evolve to the equilibrium *E*_4_{1, 1, 1}. The IESSs of the formal leader, the informal leader, and the ordinary employee are {empowering}, {emerging}, and {following} respectively. The formal leader entrusts the informal leader with some decision-making power and the informal leader can also ‘take better decisions’ than the formal leader in certain circumstances. The ordinary employee adopts the {following} strategy and works hard, and the team performance is optimal.

However, the parameters in Fig 5 are only rough values that conform to certain relationships, which cannot be a guide for accurate strategies in reality. They are adopted to demonstrate the feasibility of *E*_4_ as the unique ESS and the rationality of the distributed leadership pattern.

## 5. Discussions

All three decision-makers directly influence the other decision-makers through their epistemological frameworks and actions, whether they are the formal leader, the informal leader, or the ordinary employee. After the decision-making dynamic replication analysis, evolution stability analysis, and numerical simulation experiments among the three decision-makers in a team, all the strategy combinations can expand to the equilibrium *E*_4_{1, 1, 1}. As a result, the IESSs of the formal leader, the informal leader, and the ordinary employee are {empowering}, {emerging}, and {following} respectively, and the team performance is optimal.

This paper suggests that formal leaders could entrust informal leaders with decision-making power and informal leaders could also ‘take better decisions’ than more experienced formal leaders in certain situation. The reasons for this are various. In the first place, the workload of formal leaders is frequently overwhelming. Informal leaders usually are in charge of fewer duties and should be able to pay more attention to their daily decisions and routines, assisting the idea that delegating decision-making may enhance organizational performance. Second, with the increased task complexity and dynamism confronting modern teams, formal leaders may possess ‘the vision’ and the skills to coordinate it throughout, while lacking the heterogeneous knowledge to decide on task-specific matters. Third, as informal leaders are closer to ordinary employees, supervising ordinary employees reduces costly communication.

Informal leaders who perform informal leadership make choices and take decisions voluntarily. Several studies have proposed that informal leaders arise when a few members achieve influence over other team members in terms of direction, motivation [54], and task behavior, with the informal leaders gaining power, status, and reputation, thereby improving their performance [4,10,51,52]. On the contrary, a few scholars have designated that individuals are not inclined to lead if the costs of leading are perceived to be high compared to the benefits [54], since the individuals who undertake such endeavors are more likely to bear more responsibilities and their qualities and capabilities are highlighted [53]. However, the fact remains that people who lead informally may not be viewed positively by ordinary employees regarding their intentions, capabilities, and qualities [53]. While various people may welcome autonomy as an opportunity for self-fulfillment, others may suffer from psychological distress, such as anxiety and fear of failure [42]. Furthermore, the more people perceive risks associated with leading, the less likely they are to claim an informal leadership identity. For example, leading will adversely affect the impressions other people hold of them, especially without a formal authority demonstrating their authority, nor does it contribute any guarantee they will be viewed positively regarding their intentions, capabilities, and qualities [52,53]. Therefore, organizations should actively encourage informal leadership development, create an excellent organizational atmosphere, and entrust informal leaders with decision-making powers, because an active informal leader could assist the formal leader in managing and supervising the ordinary employees, which is a significant factor affecting optimal team performance.

For ordinary employees, it is frequently stated that there are no leaders without followers [39,43,47]. Followership behaviors include how followers approach solving problem with leaders, how they approach taking responsibility for leaders, etc. As a social learning perspective would propose, team members look to their formal leader to determine appropriate behavior within the team, and follow that behavior [37]. In addition, the sociological analysis points out that when the leaders can satisfy the followers’ psychological needs for safety and order, belonging and team, as well as work and meaning, employees may choose to follow [43,48,49]. For example, people follow leaders to satisfy their need for safety or security, so when they feel insecure or threatened, they associate themselves with a leader to reduce their anxiety. These arguments are consistent with prior research showing that by employing the perspective of instrumental rationality, it could be seen that followership could yield benefits for employees or could help fulfill their personal goals, or when employees face penalties or potential risks for not following the leaders, they will follow based on a ‘benefit-cost’ calculation [39]. It is worth mentioning that, while there is a positive correlation between leaders’ incomes and team performance [15], a few employees fail to employ team performance as their only criterion for determining their income and even engage in behaviors that undermine team performance. It has been found that increasing bonuses and reducing costs could assist the game model to reach a stable state more rapidly, so it is crucial to promote employees’ following behavior to enhance teamwork.

## 6. Conclusions

A tripartite evolutionary game model is presented in this study to study the relationship of the formal leader, the informal leader, and the ordinary employee in a distributed leadership pattern. An evolution analysis is performed from the individual perspective and the holistic perspective respectively to investigate the role of each key parameter in the evolution process. In addition, the stability of the equilibrium strategy of the evolutionary game is discussed. Simulated results demonstrate that a unique equilibrium could arise between a formal leader, an informal leader, and an ordinary employee when certain conditions are met. This equilibrium is the ideal state of distributed leadership. It is verified that positive incentive policies and strict penalties policies could make the evolutionary game system converge to desired stability faster with significant implications for team performance. In this study, the formal leader played an essential role in supporting informal leadership, which is consistent with previous research demonstrating that informal leaders are critical for teams’ ability to adjust to dynamic environments. At the same time, formal leaders should contribute adequate assistance to informal leaders. Our study echoes the previous study and contributes another perspective to highlight the importance of formal leadership and the assistance they contribute. Moreover, this study also examines the intertwined dynamics of formal and informal leadership from employees’ perspectives in organizations [15,41,42].

This study has several implications for human resource management in organizations. The widespread implementation of distributed leadership patterns is currently the need to deal with the flattening of the organization. This study asserts that its novelty is that analyzing genuine-time naturally occurring interaction illustrates how distributed leadership could be achieved in social practice. From a practical perspective, the findings of this study could inform formal leaders to switch from supervisory roles to facilitators and place a primary emphasis on identifying employees with leadership potential and developing their leadership skills. Informal leaders hope to acquire psychological trust and assistance from their peers in their leadership, less emergence risk, and more autonomy in decision-making. Ordinary employees hope to acquire material assistance such as salary raises, reducing their workloads, and mentoring from their formal leaders. Consequently, the formal leader’s responsibility is to provide assistance and energize the informal leaders and ordinary employees. On the one hand, formal leaders need to protect the informal leaders by providing necessary resources and mentorship. On the other hand, they also need to educate and communicate with employees about being a loyal follower [1]. We anticipate our research findings to provide various implications for leadership development in organizations.

This study sheds light on the tripartite game of team members, which has not yet been extensively discussed. The most significant contribution of this study is to utilize the methodology of the tripartite evolutionary game to the micro organizational behavior and contribute various insights for the organization research. It should be designated that this study possesses various limitations that open avenues for further research. In the first place, to simplify the model, it was assumed that the players’ roles were fixed at the outset. However, in the actual organization, leaders’ and followers’ identities could shift among team members through a social construction process. Next, the variable design in this study is based on the assumptions of common scenarios, but no specific variable data were collected from genuine cases, and there are inevitably other variables that are not considered, the universality of the research conclusion needs to be enhanced. Thirdly, our research is assumed in a context in which formal leaders possess power over team members. However, there may be a situation in which informal leadership is over-emergence in a distributed leadership pattern. Both formal and informal leaders try to display team leadership, so the formal leader in a team might engage in power struggles with the informal leaders. Future studies should investigate formal-informal leadership interactions in distributed leadership patterns under different contexts.

## Supporting information

S1 Program Maple program for obtaining the eigenvalues of the 7th equilibrium. (MW)

S2 Program Matlab program for plotting Fig 5. (M)
